# Next-generation probiotics – do they open new therapeutic strategies for cancer patients?

**DOI:** 10.1080/19490976.2022.2035659

**Published:** 2022-02-15

**Authors:** Karolina Kaźmierczak-Siedlecka, Karolina Skonieczna-Żydecka, Theodore Hupp, Renata Duchnowska, Natalia Marek-Trzonkowska, Karol Połom

**Affiliations:** a Department of Surgical Oncology, Medical University of Gdansk, Gdańsk, Poland; b Department of Biochemical Sciences, Pomeranian Medical University in Szczecin, Szczecin, Poland; c International Centre for Cancer Vaccine Science, University of Gdansk, Gdansk, Poland; d Institute of Genetics and Cancer, University of Edinburgh, Edinburgh, Scotland, UK; e Department of Oncology, Military Institute of Medicine, Warsaw, Poland; f International Centre for Cancer Vaccine Science University of Gdańsk, Gdańsk, Poland; g Laboratory of Immunoregulation and Cellular Therapies, Department of Family Medicine, Medical University of Gdańsk, Gdańsk, Poland

**Keywords:** Gut microbiota, next-generation probiotics, *Faecalibacterium prausnitzii*, *Akkermansia muciniphila*, *Bacteroides fragilis*, cancer, immunotherapy

## Abstract

Gut microbiota and its association with cancer development/treatment has been intensively studied during the past several years. Currently, there is a growing interest toward next-generation probiotics (NGPs) as therapeutic agents that alter gut microbiota and impact on cancer development. In the present review we focus on three emerging NGPs, namely *Faecalibacterium prausnitzii, Akkermansia muciniphila*, and *Bacteroides fragilis* as their presence in the digestive tract can have an impact on cancer incidence. These NGPs enhance gastrointestinal immunity, maintain intestinal barrier integrity, produce beneficial metabolites, act against pathogens, improve immunotherapy efficacy, and reduce complications associated with chemotherapy and radiotherapy. Notably, the use of NGPs in cancer patients does not have a long history and, although their safety remains relatively undefined, recently published data has shown that they are non-toxigenic. Notwithstanding, *A. muciniphila* may promote colitis whereas enterotoxigenic *B. fragilis* stimulates chronic inflammation and participates in colorectal carcinogenesis. Nevertheless, the majority of *B. fragilis* strains provide a beneficial effect to the host, are non-toxigenic and considered as the best current NGP candidate. Overall, emerging studies indicate a beneficial role of these NGPs in the prevention of carcinogenesis and open new promising therapeutic options for cancer patients.

## Introduction

Gut microbiota-related aspects in cancer patients have been intensively analyzed in multiple studies. The link between gut microbiota imbalance (referred to as so called dysbiosis) and development of cancers has been documented. However, the bacterial gut microbiota itself is not only altered, but also its fungal part (known as mycobiota).^1^ Gut microbiota signatures may be different depending on the types of cancer. For instance, in the case of pancreatic cancer, oral microbiota dysbiosis (differential abundance of *Porphyromonas gingivalis, Fusobacterium, Neisseira elongata, Streptococcus mitis, Bacteroides, Lepotrichia, Grabulitacetlla adiacens, Aggregatibacter actinomycetemocomitans*) and intrapancreatic microbiota changes (altered counts of *Gammaproteobacteria, Fusobacterium, Escherichia coli, Bifidobacterium pseudolongum*) hve been observed.^2^ Notably, certain bacteria and fungi may trigger the development of cancer *via* multiple mechanisms. For instance, *Escherichia coli* causes the over-proliferation of normal epithelial cells,^3^ *Enterococcus faecalis* destroys DNA *via* free radicals^4^ and *Helicobacter hepaticus*, similarly as *Trichosporon* fungal genus, increases the production of pro-inflammatory cytokines (IL-1β, IL-6, IL-8, TNF-α, and IFN-γ).^5–7^

Gut microbiota has been shown to impact anti-cancer treatment efficacy and patients’ quality of life. The microbiotome may also be used as noninvasive predictive biomarkers for early detection of cancers, for instance pancreatic cancer and hepatocellular carcinoma.^8,9^ There is a strong need to alter the composition of the gut microbiota and consequently to restore its balance to achieve better effects of multi-modal anti-cancer treatments. Prebiotics, probiotics, synbiotics, postbiotics, and fecal microbiota transplantation are being used to modulate gut microbiota and provide beneficial effects.^3,10^ Recently, Kaźmierczak-Siedlecka et al. described a randomized, double-blind and placebo-controlled study, where it was shown that a 4 week administration of a probiotic strain – *Lactobacillus plantarum* 299 v (in dose 2 × 10^10 CFU daily) in cancer patients receiving home enteral nutrition, may improve the level of albumin and importantly reduce gastrointestinal symptoms which are complications caused by enteral nutrition.^10^

The usage of probiotics in many conditions has been intensively analyzed and it is quite well established. Notwithstanding, there is still a need to search for other therapeutic strategies for cancer patients.^11^ Therefore, the identification of next-generation probiotics (NGPs) using next generation sequencing techniques and bioinformatics tools opens new options in the aforementioned context.^11–13^ Currently, data regarding NGPs and cancer patients remains undefined and limited.^3^ The definition of NGPs states that these are “live microorganisms identified on the basis of comparative microbiota analyses that, when administered in adequate amounts, confer a health benefit on the host”.^14^ Notably, “traditional probiotic strains” were isolated from gut and traditional fermented foods. By contrast, NGPs have been recently isolated using new tools allowing isolation, identification, and modification of commensal bacterial species.^3,14^ According to recently published data, NGPs groups include mainly *Faecalibacterium prausnitzii, Bacteroides fragilis, Akkermansia muciniphila, Prevotella copri, Bacteroides thetaiotaomicron, Christensenella minuta*, and *Parabacteroides goldsteinii*.^11,12,15^ However, most of them are not related to cancer/or their properties were not confirmed/investigated yet in relation to the cancer phenotype. Therefore, in our present review, we focus only on three NGPs candidate; *Faecalibacterium prausnitzii, Akkermansia municiphila*, and *Bacteroides fragilis* due to their identified linkage to oncology. We describe the characteristics of these NGPs and discuss their possible administration, efficiency, and safety in cancers.

### Faecalibacterium prausnitzii

*F. prausnitzii* is an anaerobic Gram-positive bacteria which belongs to the Firmicutes phylum and the family *Ruminococcaceae*.^11,16^ It represents more than 5% of the total bacterial population in healthy adults.^17^ *F. prausnitzii* ferments glucose and produces short-chain fatty acids (SCFAs), formic acid as well as d-lactate^11,18^ and it is the most important butyrate-producing bacteria. Butyrate – a type of SCFAs – plays a significant role^19,20^ in part as a source of energy for colonocytes. Additionally, butyrate enhances epithelial barrier integrity and mucosal immunity.^19,21^ Butyrate might also regulate the gut-brain axis.^22^ Moreover, butyrate regulates the expression of various genes through its function to inhibit indirectly the deacetylation of histones; and for instance genes encoding lipids as well as those which are associated with inflammation, differentiation, and apoptosis.^23^ Recently, it was assumed that also *Oscillospira* can produce all SCFAs (mainly) and it may be consider as a NGP candidate.^24^

*F. prausnitzii* has anti-inflammatory properties, which has been confirmed in colitis animal model studies.^25,26^ In Zhou et al., experimental colitis models were used to show that butyrate produced by *F. prausnitzii* maintains Th17/Treg balance providing anti-inflammatory effects.^27^ Moreover, *F. prausnitzii* ameliorates colorectal colitis through inhibiting histone deacetylase 1.^27^ Interestingly, not only does *F. prausnitzii* synthesized butyrate have anti-inflammatory properties, but a 15 kDa protein also mediates this effect.^28^

The abundance of *F. prausnitzii* depends on nutritional factors. In Verhoog et al., a systematic review including 29 trials and 1444 participants (5 trials regarding *A. muciniphila* and 19 – *F. prausnitzii*), it was shown that some dietary factors may modulate the abundance of these bacterial species.^29^ Mainly, a caloric restriction diet and supplementation with pomegranate extract, resveratrol, sodium butyrate, polydextrose, yeast fermentate, and inulin increased the abundance of *A. muciniphila*; in case of *F. prausnitzii* the abundance was modulated predominantly *via* prebiotics.^29^ Inulin can increase the level of *F. prausnitzii*.^16,30^ The administration of Xylo-oligosaccharide also positively affects the abundance of *Faecalibacterium* sp. and *Akkermansia* sp.^16^

Lopez-Siles et al. have shown that the abundance of *F. prausnitzii* is lower in patients with colorectal cancer, Crohn’s disease, and ulcerative colitis in comparison to healthy controls (*P* < .001).^31^ Similarly, the reduced counts of *F. prausnitzii* in colorectal cancer patients was also confirmed in Palmisano et al. study.^32^

Recently, the association between non-small-cell lung cancer (NSCLC) and butyrate-producing bacteria was also found.^33^ This study included 30 NSCLC patients and 30 healthy participants. In NSCLC group the reduced amount of butyrate-producing bacteria, such as *F. prausnitzii, Clostridium leptum, Clostridial cluster I, Ruminococcus* spp., *Clostridial Cluster XIVa*, and *Roseburia* spp. was noted.^33^ However, the mechanisms by which they may affect the development of NSCLC has not been investigated yet.

Gastrointestinal mucositis affects around 50% of cancer patients and is a complication of chemotherapy and radiotherapy.^34^ In a systematic review by Touchefeu et al. it was revealed that alterations of gut microbiota during anti-cancer treatment can occur. The decrease of *Bifidobacterium, Clostridium cluster XIVa, F. prausnitzii* and elevation of *Enterobacteriaceae* and *Bacteroides* were noted.^34^ These alterations contributed to occurrence of gastrointestinal mucositis and diarrhea. The administration of probiotics and thus restoration of gut microbial homeostasis may reduce the risk of these complications. Interestingly, Lapiere et al. assessed whether *F. prausnitzii* prevents the acute breakdown of the colonic epithelial barrier in a preclinical model of pelvic radiation disease.^35^ After radiotherapy (even more than 10 years) patients may develop diarrhea, constipation, abdominal pain, and bloating. These symptoms were recognized in 2010 and called pelvic radiation disease. In Lapiere et al. study, rats [male SD (Sprague Dawley), Janvier SA, Le Genest St Isle, France weight: 250–300 g] were locally irradiated at 29 Gray (dose of irradiation) in the colon. They received *F. prausnitzii* strain A2-165 (DSMZ collection, Braunschweig, Germany, DSM No 17677) 3 days before the irradiation and up to 3 day after that. It was noted that the administration of this NGP limited radiation-induced para-cellular hyperpermeability and the infiltration of neutrophils (MPO+ cells) in the colonic mucosa. The increase in IL-18 production by colonic crypt epithelial cells was also observed. Summarizing, these striking results suggest that *F. prausnitzii* may protect the epithelial colonic barrier from irradiation.^35^

Recently, Ma et al. also showed that *F. prausnitzii* suppressed breast cancer (BC) cell growth *via* inhibiting the IL-6/STAT3 pathway.^36^ The abundance of *Faecalibacterium* was reduced in breast cancer patients and it was negatively correlated with various phosphorylcholines. In this context, the gut microbiome may be considered as a new biomarker to detect breast cancer.^36^ Several studies in BC patients have shown that overweight and obesity women have a decrease in the total number of *F. prausnitzii* comparing patients of normal weight.^37–39^ On the other hand, obesity is an important risk factor for BC especially in postmenopausal women.^40,41^ Moreover, Goedert et al. showed that postmenopausal women with BC had altered fecal microbiota and lower alpha diversity.^42^ Further, it has been demonstrated that a subset of microbes within the gastrointestinal tract (collectively referred as estrobolome) influences estrogen metabolism and the balance of circulating and excreted hormone levels.^43,44^ Therefore, the intestinal microflora may affect the onset of breast cancer through estrogen-dependent signaling pathways.^36^ Ma et al.^36^ showed that *F. prausnitzii* was decreased significantly in breast cancer women and it may be related to its development. It seems that *Faecalibacterium* and flora metabolites such as phosphorolcholine could be useful in breast cancer detection. In preclinical model, *F. prausnitzii* found to suppress the growth of breast cancer through the inhibition of IL-6/STAT3 pathway.^36^

Gut microbiota may be also used as a prognostic biomarker to assess overall survival (OS), as demonstrated by Wei et al.^45^ High abundance of *F. prausnitzii* was related to better OS in colorectal cancer patients after a surgical procedure; by contrast, high counts of *Bacteroides fragilis* and *Fusobacterium nucleatum* were associated with worsened OS. Notwithstanding, not only the abundance of a particular bacteria/fungi have been significant in this context, but also the gut microbiota diversity. In Taur et al., a group of 80 patients undergoing allogenic hematopoietic stem cell transplantation (allo-HSCT) was divided into 3 groups, i.e. presenting low, intermediate, and high level of gut microbiota diversity.^46^ It was shown that mortality outcomes were significantly worse in participants with lower intestinal diversity. Notably, OS at 3 years after an allo-HSCT procedure was 36% for group with low microbial diversity, 60% (intermediate), and 67% for high (*P* = .019, log-rank test).^46^ Therefore, these results suggest that maintaining an appropriate microbial diversity may prolong OS in patients receiving allo-HSCT.

### Akkermansia muciniphila

*A. muciniphila* belongs to the *Verrucomicrobia* phylum. It is Gram-negative and oval-shaped bacteria which was discovered as the first member in the genus *Akkermansia*.^47–49^ Despite the fact that this bacteria belongs to the *Verrucomicrobia* phylum, the similarity between both genomes is very small. *A. miciniphila* was discovered in 2004 at Wageningen University of the Netherlands (Muriel Derrien’s Ph.D. thesis),^50,51^ whilst searching for a new mucin‐degrading microbe in human feces.^50,52^ Originally, *A. muciniphila* was isolated from a fecal sample from a healthy female in a specific medium containing purified mucins and a sole carbon source.^47^ *A. muciniphila* can be detected using 16S rRNA gene sequencing.^53^ Importantly, pasteurized *A. muciniphila* is the first NGP providing beneficial effects that was approved by EFSA.^54^ The consumption of 3.4 × 10^10^ cells/day is safe for the target population whereas amount of viable cells in novel food is less than 10 CFU/g.^54^

*A. muciniphila* has been assessed as an aerotolerant anaerobic bacterium which colonizes the mucus layer of the human gastrointestinal tract.^47,52^ The largest amounts of this bacteria is located in the colon, however, it can be also found in other parts of gastrointestinal tract.^55^ *A. muciniphila* is able to grow in a wide range of temperatures, i.e. 20–40°C (the optimum growth at 37°C) and in pH values ranging from 5.5 to 8.0 (optimum – 6.5).^50,55^ Moreover, it can tolerate low levels of oxygen (nM concentrations) and is able to grow in the presence of 0.1% purified bile salts.^55^ *A. muciniphila* encodes 567 secreted proteins, for instance sugar hydrolase, sialidase, and sulfatase. Fecal microbiota contains 1–4% of *A. muciniphila*.^47,56^ *A. muciniphila* colonizes the human gut within 1 year after birth and its level remains stable in healthy adults. However, the abundance of this bacteria has been found to gradually decrease with older age.^52^ *A. muciniphila* counts depend on dietary factors and its abundance increases in the intestinal tract supplied with polyphenols, which can be found in cereals, vegetables, coffee, tea, grapes, cranberry, and wine.^16^

*A. muciniphila* provides several beneficial effects to humans. It regulates metabolic pathways through affecting glucose tolerance and lipid metabolism.^51,57^ The latest evidence states that daily oral administration of pasteurized *A. muciniphila* alleviates diet-induced obesity and decrease food energy efficiency^58^ and the mechanism behind this might include reduction of carbohydrate absorption and enhanced intestinal epithelial turnover. According to some data, reduced levels of *A. muciniphila* was found in patients with diabetes, obesity, hypertension, liver diseases, intestinal inflammation, and IBDs (ulcerative colitis, Crohn’s disease).^53,55–64^ *A. muciniphila* may be effective in supporting the treatment of obesity-related disorders including cardiometabolic diseases.^65^

*A. muciniphila* maintains intestinal immunity and regulates gut barrier functions. It improves mucus thickness through regulating zonula occludens-1, occludin, and claudin 3.^65^ Additionally, *A. muciniphila* restores the amount of Goblet cells and increases mucin-2 expression.^16^ *A. muciniphila* is able to prevent the development of metabolic endotoxemia.^66^ Ottman et al. identified a highly abundant outer membrane pili-like protein of *A. muciniphila* MucT, which modulates the host immune response as well as gut barrier integrity.^67^ It was noted that *A. muciniphila* enhances the immune system through regulation of specific cytokines and acts *via* Toll-like receptors (TLRs), such as TLR2 and TLR4.^67^ *A. muciniphila* acts against pathogens. It reduces inflammation induced by *Porphyromonas gingivalis*,^68^ which is an opportunistic oral pathogen causing periodontitis and participating in carcinogenesis of pancreatic and esophageal cancers.^69,70^ Moreover, Huck et al. observed that *A. muciniphila* increased the expression of integrin-β1, E-cadherin and ZO-1 in TIGK cells, and also confirmed its role in maintaining junctional integrity.^68^

The abundance of *A. muciniphila* is regulated by dietary factors. The up-regulation of this bacteria may also be obtained *via* the Huoxue Yiqi Recipe-2 (HYR-2), which came from the Ze Qi Decoction in one of the four great classics of Traditional Chinese Medicine (TCM) called “Synopsis of Prescriptions of the Golden Chamber”.^71^ HYR-2 down-regulates the expression of PD-L1, which might be related to the blocking effect of HYR-2 on the PI3K/Akt signaling pathway. Overall, HYR-2 plays an anti-lung cancer role by regulating PD-L1 and the level of *A. muciniphila*.^71^ According to recently published data, there is a link between gut microbiota, probiotics, NGPs and immune system as well as immunotherapy efficacy.^72–74^ *A. muciniphila* has an impact on the effect of immunotherapy based on anti-PD-1 agents. In Xu et al. mice model study it was noted that *A. muciniphila* affects the metabolism of glycerophospholipid and consequently maintains the anti-PD-1 antibody.^75^ Moreover, in another study, Routy et al. have shown that cancer patients treated with anti-PD-1/PDL-1 antibodies lived significantly shorter if they also had received oral treatment with antibiotics.^76^ Interestingly, the response to this therapy was related to the abundance of *A. muciniphila*. Additionally, both transplantation of the microbiota from patients responding to immune checkpoint inhibitors (ICIs) and supplementation with this NGP alone restored the sensitivity to immunotherapy.^76^ Similarly, the results of another study demonstrated that abundance of *A. muciniphila* is associated with clinical response to anti‐PD‐1‐based immunotherapy in metastatic melanoma patients.^77^ The combination of IL-2 and *A. muciniphila* may restore IL-2-based immunotherapy efficacy.^78^ This combination enhances anti-tumor immune responses through recruiting tumor-specific cytotoxic T lymphocytes and decreasing immunosuppressive Tregs within the tumor microbiota.^78^

The gut microbiome and metabolome may be altered in lung cancer patients. Recently, it was shown that the abundance of commensal bacteria, such as *A. muciniphila, Rikenellaceae, Bacteroides, Peptostreptococcaceae, Mogibacteriaceae*, and *Clostridiaceae* were diminished in patients with NSCLC compared to controls.^79^ Chen et al. indicated that *A. muciniphila* enhances the antitumor effect of cisplatin (CDDP) in Lewis lung cancer mice.^80^ In this study, 50 mice were divided into 5 groups (i.e. normal, model, CDDP, CDDP + *A. muciniphila*, and CDDP + antibiotics) and they were treated for 5 weeks. Among others, in CDDP + *A. muciniphila* groups, downregulation of the ki-67, p53, and factor-associated suicide (Fas) ligand proteins levels were noted. The expression of CD4+ CD25+ Foxp3+ Treg’s was also suppressed in the peripheral blood and spleen of mice. Additionally, the levels of IFI27l2 and IGFBP7 were increased. CDDP supplemented with *A. muciniphila* may be a first line treatment in lung cancer. It opens a novel promising therapeutic strategy for lung cancer patients.^80^

There are also identifications of additional factors expressed from *A. muciniphila*. Amuc_1434* is a protein derived from *A. muciniphila* which suppresses LS174T cell viability, the mitochondrial pathway of apoptosis by up-regulating tumor-necrosis-factor-related apoptosis-inducing ligand, and as a consequence it inhibits development of colorectal cancer.^81^ Interestingly, the pasterization process does not affect the biological activity of the pilli protein.^58^ The toxicological safety evaluation of this probiotic was done recently in 2021. In Druart et al. study, Han Wistar rats received orally *A. muciniphila* in doses of 75, 375, or 1500 mg/kg body weight/day for 90 days.^82^ No adverse events after administration of *A. muciniphila* were noted and the authors concluded that this probiotic is safe as a food ingredient.^82^ However, it may promote colitis, which was shown by Seregin et al. in mice model studies.^83^

Fruge et al.^84^ have shown differences in gut microbiota related to elevated body fat, highlighting the prevalence of *A. muciniphila* in stage 0–II breast tumors. Additionally, in BC women with high relative abundance of *A. muciniphila*, higher abundance of *Prevotella* and *Lactobacillus* and lower of *Clostridium, Campylobacter* and *Helicobacter* were detected when compared to patients with low relative abundance of the bacteria.^84^

### Bacteroides fragilis

*B. fragilis* is defined as a commensal, Gram-negative obligate anaerobe, which resides in the lower part of the human gastrointestinal tract. It constitutes around 1% of gut microbiota.^85–87^ However, there is body of evidence proving its abundance in mouth, upper respiratory tract and female genital tract. This genus is widely known an opportunistic pathogen, implicating the elevation in gut barrier permeability thus associated with colitis and to at least some extent systemic inflammation.^88–90^ These are associated with *bft* genes encoding *B. fragilis* toxin in pathogenicity Islands (BfPAI).^91^ Enterotoxigenic *B. fragilis* toxins (EBFTs) also contribute to tumor formation through activation of STAT3 and Wnt pathway as well as stimulation of IL-17 production.^3^

The latest evidence, however, indicates that nontoxigenic *B. fragilis* strains might exert probiotic properties. Apart from typical mechanisms of action maintaining gut homeostasis, Polysaccharide A (PsA) and other outer membrane vesicles delivering certain beneficial molecules of this NGP have been reported to affect positively gut health.^92^ It is of the major importance that its counts elevates along with the development of the immune system of a child, between 1 and 2 years of age.^93^

Traditionally, *B. fragilis* interferes with other microbes *via* inhibiting their growth or translocation. In a Deng et al. animal model study, *B. fragilis* was assessed in the prevention of *Clostrioides difficile* infection (CDI).^94^ The CDI mouse (*C. difficile* strain VPI 10463 spores) were prophylactically supplemented with *B. fragilis* and it was shown that treatment with this probiotic strain improved bacterial diversity and was associated positively with abundance of *A. muciniphila. B. fragilis* inhibited *C. difficile* adherence via prevention of apoptosis as well as zonula occludens-1 (ZO-1) and (mucin-2) MUC-2 loss. Consequently, *B. fragilis* maintained intestinal barrier integrity.^94^ In another study, it was noted that *B. fragilis* culture inhibits the translocation of *Salmonella* Heidelberg.^87^ This competitive properties are due to secretion of antimicrobial protein-1 (BSAP-1) containing membrane attack/perforin (MACPF) domains lysing bacterial cells or infecting host cells.^95^ Another protein involved in such competition might be eukaryotic-like ubiquitin protein (BfUbb).^95^ Of note, contact-dependent Type VI secretion system (T6SSs) has been also found to play a role in this antagonism.^96^ At last, studies have shown that *B. fragilis* produced short chain fatty acids and inhibited the growth of pathogens and are able to elevate Tregs counts.

Apart from competitive properties, majority of beneficial actions of nontoxigenic *B. fragilis* NCTC 9343 is due to PSA. It was proved that PSA of *B. fragilis* NCTC 9343 – delivered *via* outer membrane vesicles – diminished the imbalance in Th1/Th2 cell counts in germ free mice and elevated Treg activity.^97^ Also, zwitterionic polysaccharides of *B. fragilis* were found to be internalized by antigen-presenting cells (APCs), and then presented on major histocompatibility complex (MHC) class II molecules affecting CD4 + T cell response.^98^ Additionally, TLR2 expression on CD4 + T cells and TLR2 signaling are important to induce IL-10 synthesis and consequently inhibition of inflammatory state.^99^

Lipopolysaccharides (LPS) are released by antibiotic administration and they increase the expression of pro-inflammatory cytokines, negatively affecting tight junctions as well as inducing development of “leaky gut”.^100^ Notably, “leaky gut” causes abdominal symptoms, such as bloating, cramps, and fatigue.^101^ *B. fragilis* also contributes to development of food allergies and sensitivities as well as multiple diseases/conditions.^101^ *B. fragilis* HCK-B3 has been isolated from healthy Chinese donors.^102^ *B. fragilis* HCK-B3 and *B. ovatus* ELH-B2 maintains gut microbiota diversity and reduces inflammation induced by LPS through both decreasing pro-inflammatory mediator, i.e. TNF-α and increasing IL-10 (anti-inflammatory cytokine) and recovering the Treg/Th-17 balance.^100^ At last, PSA *B. fragilis* NCTC 9343 immunization might reverse non-responsiveness to CTLA-4 blockage therapy in cancer patients.^102^

Overall, *B. fragilis* functions by multiple mechanisms which includes its interaction with other microbes, restoring gut microbiota balance as well as maintaining mucosal immunity and gut barrier integrity state this bacteria genus as probiotic. The safety evaluation of *B. fragilis* HCK-B3 was conducted by Tan et al.^103^ No intracorporal pathogenic properties were observed regarding body weight, hematological parameters (neutrophils, lymphocytes, hemoglobin, platelets), liver parameters (triglyceryde, cholesterol, aminotransferase), cytokines production, and tissue integrity. The adverse events after administration of *B. fragilis* HCK-B3 were rarely noted in healthy and immune-deficient mice. These results have indicated that the potential NGP strain, *B. fragilis* HCK-B3, is non-toxigenic and safe.^103^ More studies evaluating safety of different *B. fragilis* strains are yet to come.

**Figure 1.**
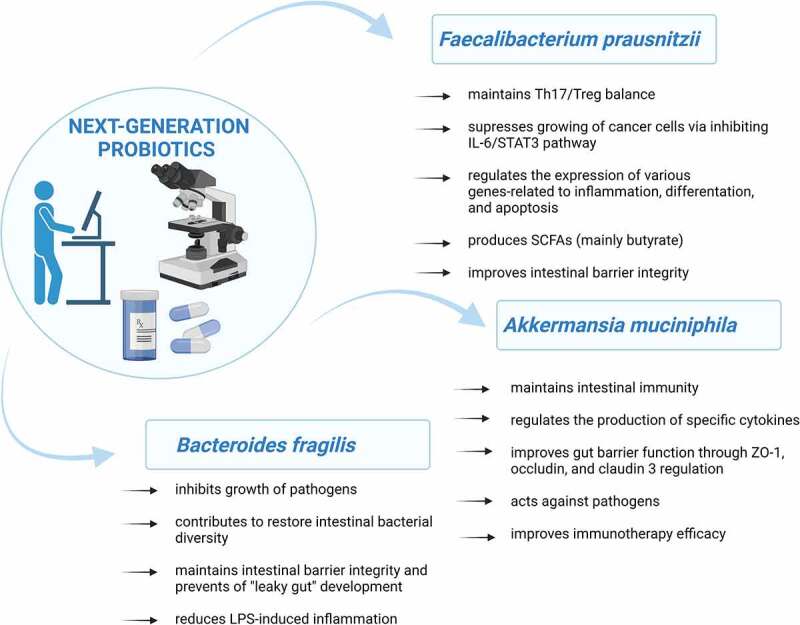
The potential mechanisms of NGPs by which they may be effective in prevention of cancer development/treatment. LPS – lipopolysaccharides, SCFAs – short-chain fatty acids, ZO-1 – zonula occludens-1. Own elaboration based on literature.^19,20,27,35,68,78,87,100^

A summary of *F. prausnitzii, A. muciniphila*, and *B. fragilis* properties in the context of oncology is presented in Figure 1.

## Conclusions

*F. prausnitzii, A. muciniphila*, and *B. fragilis* belong to the NGPs group and can be useful in cancer patients through several mechanisms. Notably, each of them exhibits different properties, however, they share similar functions and mechanisms of action. They were demonstrated to enhance the immune system, reduce LPS-related signaling, improve the activity of gut microbiota, and prevent the development of leaky gut *via* maintaining intestinal barrier integrity. Additionally, *F. prausntizii* can be effective in reduction of gastrointestinal complications caused by chemotherapy/radiotherapy whereas *A. muciniphila* may improve the efficiency of immunotherapy.

The safety of these NGPs in human cancer patients remains unclear and needs to be established more precisely. Accordingly, to some data, they are non-toxigenic and safe. Notwithstanding, *A. muciniphila* may promote colitis, which was indicated in an animal model study. Additionally, enterotoxigenic *B. fragilis* stimulates chronic inflammation and may contribute to development of colitis and colorectal cancer.

Currently, studies regarding NGPs are ongoing worldwide. In *ClinicalTrials.gov* system there are registered trials regarding *A. muciniphila* (*ClinicalTrials.gov* identifier: NCT04797442, NCT02637115) and *F. prausnitzii* (e.g., NCT04938843, NCT02538354); however, they are not related to cancers aspects (till July 2021). Most of them are associated with metabolic disorders and Crohn’s disease. Despite the fact that data regarding NGPs and cancers is still strongly undiscovered and limited, some studies indicate their beneficial role in supporting anti-cancer management, thus also open a new promising options in oncology.
